# Cold atmospheric plasma deposition of antibacterial polypyrrole–silver nanocomposites on wearable electronics for prolonged performance†

**DOI:** 10.1039/d4tc00844h

**Published:** 2024-07-05

**Authors:** Ulisses Heredia-Rivera, Akshay Krishnakumar, Venkat Kasi, Muhammad Masud Rana, Sarath Gopalakrishnan, Sina Nejati, Gagan Gundala, James P. Barnard, Haiyan Wang, Rahim Rahimi

**Affiliations:** a School of Materials Engineering, Purdue University West Lafayette IN 47907 USA rrahimi@purdue.edu; b Birck Nanotechnology Centre, Purdue University West Lafayette IN 47907 USA; c School of Electrical and Computer Engineering, Purdue University West Lafayette IN 47907 USA

## Abstract

Wearable electronics have become integral for monitoring physiological parameters in diverse applications, particularly in medical and military fields. e-Textiles, featuring integrated conductive threads or fabrics, offer seamless integration and comfort for prolonged contact with the body. Despite their potential, the biofouling of textile-based electrode systems by skin microbes remains a significant challenge, limiting their operational lifespan. Recent studies have highlighted the efficacy of conductive nanocomposites with antibacterial agents, such as silver nanoparticles (AgNPs), in addressing biofouling concerns. However, implementing such systems on 3D fibrous structures and textile surfaces often proves complex and inefficient. To overcome these challenges, we explored cold atmospheric plasma (CAP)-based *in situ* polymerization for the direct deposition of functional conductive polypyrrole–silver (PPy–Ag) nanocomposites onto conductive textile surfaces. For this process, a customized CAP deposition system was engineered, enabling precise material deposition through robotic control of the plasma jet. This process achieved direct, conformal attachment onto textile fibrous structures, ensuring uniform distribution of conductive polypyrrole and silver in the form of AgNPs throughout the polymer polypyrrole matrix without compromising fabric flexibility and breathability, which was validated through different surface electron microscopy and chemical analysis (*e.g.*, EDX, FTIR, Raman, and XRD). Systematic studies with various precursor mixtures identified an optimized PPy–Ag composition that demonstrated stable antibacterial properties and biocompatibility against common skin microbes and epithelial cells. Systematic studies with various precursor mixtures identified an optimized PPy–Ag composition, with the precursor mixture containing 96 wt% pyrrole and 4 wt% AgNO_3_ weight ratios as the optimal surface coating process, demonstrating stable antibacterial properties and biocompatibility against common skin microbes and epithelial cells respectively. As a proof of concept, the nanocomposite coating was applied to conductive carbon fabric surfaces as dry electrodes in a wearable garment for continues electrocardiography (ECG) monitoring over 10 days. Results revealed a significantly longer performance of the dry electrodes as comparable to standard gel-based Ag/AgCl electrodes (1 day) while providing less noise in ECG signal measurements from the subject, showcasing the potential of this technology for practical wearable applications. Envisioned as a groundbreaking solution, this technology opens new avenues for the scalable and effective integration of functional conductive circuits and sensors into everyday garments, ensuring prolonged and efficient performance in wearable electronics.

## Abstract 2

This study demonstrates the use of Cold atmospheric plasma-assisted deposition of conductive PPy–Ag nanocomposite coatings onto fabric electrodes, for enhancing the long-term antibiofouling performance of e-textile-based electronics.

## Graphical abstract

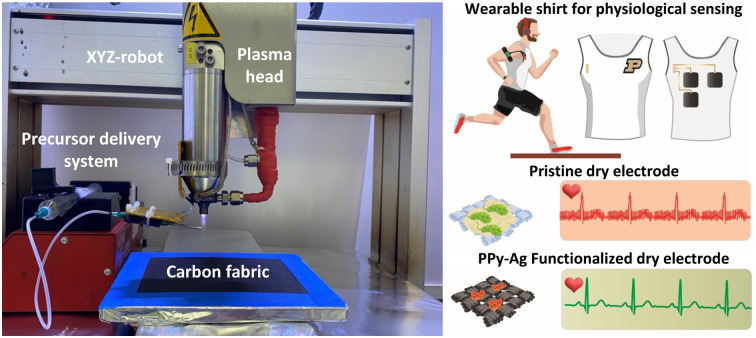

## 1. Introduction

The transformative trajectory of wearable electronic technology, with a growing interest in commercialization, is experiencing widespread adoption in daily life.^1^ This extends from smartwatches to more advanced healthcare monitoring gadgets. With a global market size of $138 billion in 2022 alone, the market is expected to grow at a compound annual growth rate of 13.60% during the forecast period 2023 to 2032.^2^ One primary area of focus in developing wearable healthcare sensors is to integrate them with everyday garments, particularly for continuous physiological monitoring in extreme situations, such as with military personnel and patients in hospital settings.^3–5^ Development of such healthcare sensors integrated into garments for physiological monitoring requires the use of a conductive surface embedded into the fabrics to act as an electrode. An effective approach to integrate such electrode surfaces seamlessly into fabric involves the utilization of conductive fabrics within the clothing, commonly referred to as e-textiles.^6^ Many efforts have been focused on integrating conductive fabrics such as carbon cloth and metallized threads into the garments for continuous monitoring of the physiological signals such as ECG, EEG, EMG *etc.*^7^ Such altercations can be embedded into the standard textile production process such as cutting, stitching, thermal bonding, and screen-printing techniques, to aid mass manufacturability and scalability. Despite the clear potential of wearable technologies for continuous health monitoring and personalized interventions, biofouling acts as a major roadblock, significantly hindering their long-term performance and reliability, leading to a progressive decline in their effectiveness over time.^8^ This insidious process involves the accumulation of biological material, such as proteins, bacteria, and other microorganisms, on the surface of these devices.^9,10^ While seemingly innocuous, biofouling can have significant consequences, negatively impacting their performance and even leading to complications. Biofouling is not a single entity, but rather a complex phenomenon driven by various factors. Skin secretions, including sweat and sebum, provide a nutrient-rich environment for microbes to thrive.^11^ Coupled with the warm, humid conditions created by the sensor's contact with the skin, an ideal breeding ground for microorganisms is established. As these microbes colonize the surface, they form a biofilm, an intricate matrix that further attracts and traps additional biomolecules.

The consequences of biofouling are multifaceted and can be detrimental to the functionality and safety of wearable sensors and electrodes.^12^ Firstly, it acts as a physical barrier, impeding the accurate transfer of bioelectrical signals, leading to distorted and unreliable data. This compromised data integrity renders the sensor ineffective in accurately monitoring crucial health parameters.^12^ Additionally, biofouling can alter the electrochemical properties of the electrodes, hindering their ability to interact with target analytes accurately, further compromising measurement accuracy. Furthermore, the presence of biofilms can create an environment conducive to the growth of pathogenic bacteria, potentially leading to skin irritation, allergies, and even infections. This raises concerns about the safety and long-term wearability of these devices. To address this challenge, researchers are actively exploring diverse mitigation strategies, categorized into four main approaches including surface modification with antibacterial agents as well as enzyme and peptide, micro- and nano-surface texturing, and electrical or mechanical stimulation.^12^

In the surface modification with antibacterial agents approach the electrode is often modified with compounds possessing inherent antibacterial properties, primarily silver derivatives.^13^ Pure silver electrodes and silver/silver chloride composites, for instance, offer high electrical conductivity and tissue compatibility, coupled with natural antibacterial characteristics that minimize bacterial attachment and biofouling. However, many of these approaches face the complications of necessitating the use of large quantities of silver which increases cost and can lead to skin irritation and localized tissue toxicity due to direct contact.^14^

The enzyme and peptide surface modification approach draws inspiration from nature and utilizes enzymes and peptides with inherent antifouling properties.^15^ While offering advantages over silver and nanomaterials, effectively immobilizing these biomolecules onto the electrode surface remains a challenge. Additionally, such modifications can alter the electrode's chemical properties, hindering effective skin contact and electrical readings.

The surface micro- and nano-structuring approach modifies the electrode surface with nano-textured features, creating a natural barrier against bacterial attachment by increasing surface roughness.^16^ These acts as a physical barrier that impedes bacterial adhesion by puncturing bacterial cell membranes and thereby hindering their ability to firmly attach to the electrode surface.^16^ However, the low adhesion of these nanomaterials to the electrode surface can lead to their gradual detachment and loss of efficacy during wear and tear. In addition, while laser etching/texturing surfaces on electrodes have demonstrated high antibacterial characteristics, they are often associated with altering both the physical and chemical characteristics of the electrode surface. This alteration can lead to a high content of oxidized elements, resulting in low electrical conductivity and a poor interface with biological tissues.^16^ Conversely, the use of electrical and mechanical stimulation to induce agitation of implant surfaces towards antibiofouling has gained significant attention in recent times. Nonetheless, frequent application of high voltages or frequencies can lead to localized changes in the skin's electrochemistry, potentially resulting in irritations.^17^

In recent years, conductive polymer-based nanocomposite coatings on electrode surfaces have gained significant traction due to their unique antibacterial and conductive properties. An intriguing nanocomposite coating for inhibiting bacterial attachment and mitigating biofouling effects involves the use of polypyrrole (PPy) in combination with silver nanoparticles (AgNP).^17^ The synergistic effect of the inherent antibacterial properties of PPy, combined with the bactericidal properties of AgNP, holds promise for application to electrode surfaces for wearable electronic applications.^18,19^ Moreover, entrapping the nanoparticles within the polymer matrix mitigates their release into the environment, thereby decreasing cytotoxicity and skin irritation. Several attempts have been made in the past to develop polypyrrole-silver (PPy–Ag) nanocomposite coatings through chemical reactions to form a slurry or solution, which are then deposited onto the targeted surface using printing or dip-coating methods.^20–23^ Despite the simplicity of synthesizing such composite materials, these processes come with numerous complications, including challenges in developing an appropriate ink composite, as well as intricate drying and sintering procedures.^24^ Additionally, the conductive polymer composites inks/pastes necessary for printing processes often require bulk solution preparation, leading to increased costs and limited shelf life for the overall process.^25^

**Fig. 1:**
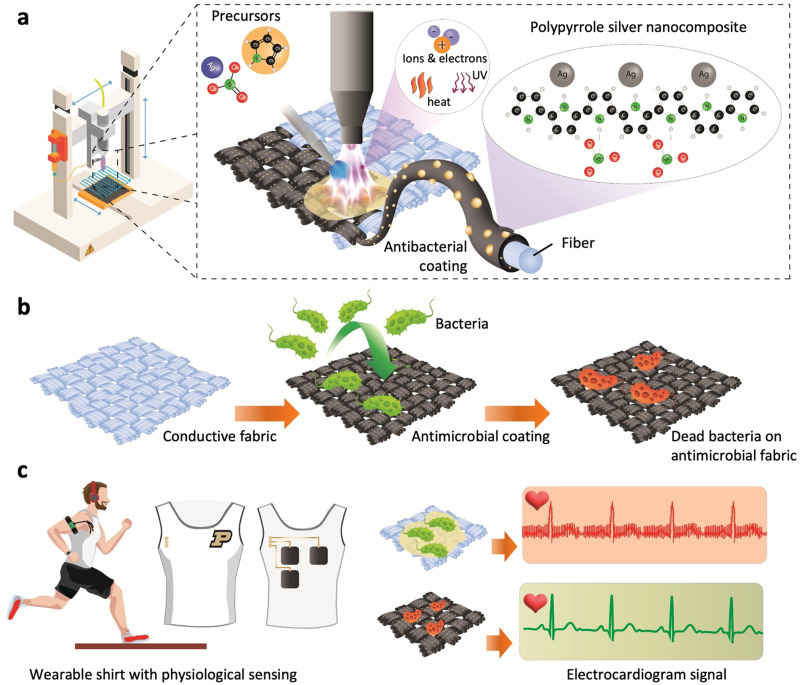
Cold atmospheric plasma-assisted deposition of conductive nanocomposite coating on fabric for wearable electronic applications. (a) Schematic representation of the selective and automated process for PPy–Ag nanocomposite coating, (b) illustration highlighting the antibacterial effect of PPy–Ag coated fabrics, and (c) schematic representation of integrated conductive fabric electrodes featuring the PPy–Ag nanocomposite coating, enabling prolonged ECG measurements with minimal biofouling.

Recently, cold atmospheric plasma (CAP) deposition technology has provided a new step into atmospheric *in situ* synthesis and direct deposition of conductive polymers and their composites onto various substrates at relatively low temperatures.^26^ This technology renders a scalable and cost-effective way of manufacturing wearable and flexible devices while reducing the cost associated with material waste during the polymer synthesis and deposition process on the targeted surface.^26^ Thus, as an effective way to enhance the longevity of wearable e-textile surfaces through the application of a conductive and antibiofouling PPy–Ag nanocomposite coating, CAP-assisted surface modification of the substrates was investigated as shown in Fig. 1. In this process carbon cloth was used as a conductive base substrate over which the PPy–Ag nanocomposite coatings were applied as a conductive anti biofouling surface modification.

A mixture of pyrrole and silver nitrate (AgNO_3_) solution was injected into a plasma torch, resulting in *in situ* fragmentation of the monomer and chain polymerization, while concurrently incorporating AgNPs into the matrix, thus forming PPy–Ag nanocomposite coatings (Fig. 1a).^26^ The highly activated pyrrole monomer reacts with the reducing Ag ions upon interaction with the reactive plasma species, leading to the formation of AgNPs inside the highly cross-linked PPy matrix.^2^ To identify the optimal proportion of Ag filler into the PPy structures that offers both high electrical conductivity and low biofouling while ensuring minimal toxicity toward skin tissue, a systematic study was conducted by varying the monomer composition.^27^ The structural, morphological, functional, elemental, and electrical properties of the deposited PPy–Ag nanocomposites were characterized using diverse characterization techniques. Antibacterial and antifouling performance of the developed PPy–Ag nanocomposite surfaces were performed using two common Gram-positive and Gram-negative skin surface flora such as *Staphylococcus epidermitis*^28^ and *Escherichia coli*,^29^ along with an assessment of biocompatibility with HCT-8 epithelial cells (Fig. 1b). Finally, in order to validate the improved longevity characteristics of the optimized PPy–Ag electrode surface modification, skin impedance and ECG signal quality measurements were performed without the use of any conductive gels or skin preparation (Fig. 1c).

## 2. Materials and methods

### 2.1 Materials

Pyrrole monomer (reagent grade, 98%) and silver nitrate (AgNO_3_, 99%) were procured from Thermo Scientific Chemicals (MA, USA). Ethylene glycol (EG) with (reagent grade, 99.8%) was purchased from Sigma Aldrich (MO, USA). All chemicals were used as received without any further modifications. In this study, plain carbon cloth (1071 HCB) procured from FuelCell (CO, USA) was utilized as the model conductive fabric substrate for the nanocomposite coating. Initially, the AgNO_3_ stock solution was prepared by dissolving 1g of AgNO_3_ in 5g of EG solution. Pyrrole monomer and AgNO_3_ stocks were mixed in their respective ratios to obtain precursor mixtures of 0.5 wt%, 1.0 wt%, 2.0 wt%, and 4.0 wt% of AgNO_3_ in pyrrole solutions.

### 2.2 CAP-assisted PPy–Ag nanocomposite coating process

All PPy–Ag nanocomposite coatings were performed by using a cold plasma atmospheric jet (Plasmatreat, PE39, NRW, Germany) coupled with a syringe pump for precursor delivery. The plasma stream was generated by delivering nitrogen as the plasma-generating gas at a rate of 30 L min^−1^, operating at a frequency of 23 kHz and a voltage of 280 V. The precursor delivery system consists of a syringe loaded with the precursor mixture and connected to a 14-gauge hypodermic needle (1.83 mm outer diameter) and placed inside the in the plasma glow. The precursors mixture was then injected into the plasma glow region, positioned 2 mm below the plasma generating nozzle, at a constant flow rate of 400 μL min^−1^. To achieve complete coverage of the substrate, the system was programmed to scan the entire substrate twice with a step size of 1 mm for line scans, a scan speed of 50 mm s^−1^, and a fixed spraying distance of 10 cm from the substrate.

### 2.3 Material characterizations

Water contact angle measurements of the modified pristine CC surfaces were performed using a Drop shape analyzer (KUSS DSA25, Hamburg, Germany) with water drops of 3 μL. Breathability of the fabric surface before and after PPy–Ag nanocomposite coatings were performed by placing 8 mm circular fabrics inside a threaded holder connected with a pressure sensor and an airflow meter. All measurements were performed delivering a continuous airflow of 1 to 3 L min^−1^ controlled *via* a flow meter. Each measurement was performed in triplicates per condition to evaluate the effect of plasma deposition on the air flow characteristics of the CC. The surface morphology and composition analysis of PPy–Ag nanocomposite coatings were characterized by performing scanning electron microscopy (SEM) and energy-dispersive X-ray spectroscopy (EDX) respectively using FE-SEM (Hitachi-S 4800, Tokyo, Japan). All SEM micrographic images were captured using an accelerated voltage of 10 kV and an emission current of 20 μA. The images were then analyzed using ImageJ software to determine the thickness of the PPy–Ag nanocomposite coatings on the CC fibers. The XRD diffraction patterns of PPy–Ag nanocomposite coatings were recorded with a PANalytical Empyrean diffractometer (Malvern Panalytical operated, MA, UK) with a voltage of 45 kV and a CuKα radiation source (*λ* = 1.544 Å) in the diffraction range of 2*θ* = 15 to 90°. The average crystallite size of PPy–Ag nanocomposite coating was calculated based on the Scherrer's equation shown below:1
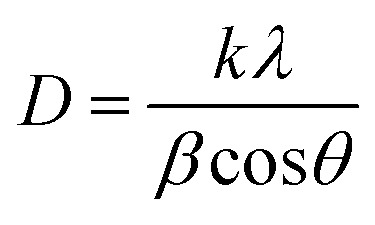
where *D* is the crystallite size expressed in nanometers (nm), *K* is the constant shape factor (0.89), *λ* is the wavelength of the X-ray radiation in nm, *β* is the full width half maximum (FWHM) of the diffraction peak and *θ* the corresponding angle in radians.^30^ The chemical structure of PPy–Ag nanocomposite coating was analyzed using attenuated total reflection Fourier transform infrared spectroscopy (ATR-FTIR) with an Id7 ATR accessory attached to a Nicolet™ iS™ 5 FTIR Spectrometer (ThermoFisher, MA, USA). Raman spectra of all the deposited surfaces were collected with the Renishaw in Via^TM^Qontor confocal Raman microscope (Renishaw plc) (RENISHAW, WD, IL).

### 2.4 Bactericidal activity

All antibacterial analysis of the pristine and PPy–Ag nanocomposite coated CC samples were performed using the common Gram-negative *Escherichia coli* (ATCC 25922, *E. coli*) and Gram-positive *Staphylococcus epidermitis* (NRS101, *S. epidermitis*) bacteria. Two comprehensive tests were performed to evaluate the antibacterial efficacy of the PPy–Ag nanocomposite surface namely, inhibition zone and contact killing analysis. Initially, the inhibition zone analysis aimed to assess the ability of the deposited surface to release antimicrobials to the surrounding environment. All bacterial strains were revived from frozen stock and cultured overnight in Tryptic Soy Broth (TSB, 30 g L^−1^) at 37 °C.^31^ After washing and resuspending the cultures in PBS, bacterial suspensions were diluted to an OD_600 nm_ of 0.25 which corresponds to ∼10^8^ CFU mL^−1^.^32^ Subsequently, 100 μL of the suspensions were uniformly spread onto a TSB agar plate, followed by the addition of 10 mm disks of pristine and PPy–Ag nanocomposite coated CC samples. The plates were then incubated at 37 °C for 16 h, and the inhibition zone around each sample were photographed and the diameters were measured.

Next, to assess the bactericidal property of the PPy–Ag nanocomposite-coated fabric surface upon bacterial attachment, a contact killing test was performed. Initially, 10 mm disks of carbon cloth and the PPy–Ag nanocomposite-coated samples were placed in a 24-well plate, followed by the addition of 100 μL of bacterial suspensions. After 24 hours of incubation, the viable bacteria from the fabric surfaces were recovered by immersing and sonicating the fabric samples in a PBS solution (pH 7). Subsequently, the samples were vortexed, serially diluted in PBS, plated on TSB agar plates, and the colonies were counted after overnight incubation.

### 2.5 Biocompatibility assessment

*In vitro* biocompatibility assessment of the pristine CC and the PPy–Ag nanocomposite-coated surfaces was conducted using HCT-8 epithelial cell lines obtained from ATCC CCL-244 (VA, USA). The cells, used between 8 to 10 passages, were cultured in Dulbecco Modified Eagle Medium (DMEM) medium supplied with 10% Fetal Bovine Serum (FBS) (Invitrogen Inc., Carlsbad, CA). Culturing was performed in a T-75 flask at 37 °C in a humidified environment (95%) with 5% carbon dioxide for 3 days until 80% confluency. Next, the cells were trypsinized, washed, resuspended, and diluted in growth medium at obtain a concentration of 5000 cells per mL before two complimentary toxicity assessments were performed. Initially, the disruption in the metabolic activity of the cell model exposed to the PPy–Ag nanocomposite coated fabric surface was analyzed using MTT cell proliferation assay *via* % cell viability. For this test, 100 μL of the diluted cell suspensions were seeded into a 96-well plate and allowed to attach to the surface for 24 h followed by the addition of the modified fabric surfaces. After subsequent 24 h, the fabric surfaces were withdrawn from the well and MTT assay cell proliferation assay was performed according to the manufacturer's instructions. Briefly, 100 μL of the MTT tetrazolium salt was added to the cells and were incubated for 3 h. Thereafter, 200 μL of the detergent solution was added to the solution, and plates were placed in a microtiter-plate shaker for 2 h at room temperature. The absorbance of the resulting solutions in the well plate was measured at 550 nm using a BMG Labtech clariostar (NC, USA).

Next, in order to visualize the viability and morphology of the cells, live/dead staining analysis was performed using LIVE/DEAD™ Viability/Cytotoxicity Kit (ThermoFisher, Massachusetts, USA). The seeding and addition of the PPy–Ag coated fabric samples followed a protocol similar to that of the MTT assay, as described previously. After 24 h of interaction, the coated fabric samples were withdrawn from the well plates followed by the addition of the fluorescent dyes to distinguish between live and dead cells based on cell membrane integrity. The fluorescent dyes were imaged using a Nikon Ti2 Eclipse microscope (NY, USA), equipped with their respective filters, under a 10× optical lens, and analyzed using NIS-Elements D software.

### 2.6 Biofouling assessments

Antifouling performance of the pristine CC and optimized PPy–Ag nanocomposite coating was investigated by immersing fabric surface in a PBS solution containing 10^8^ CFU mL^−1^ of *E. coli*. The electrode surfaces were successively immersed in a solution containing bacteria and incubated at 37 °C for 24, 48, and 72 h with gentle shaking. After washing with gentle rinse, EIS measurements were performed in 10 mL of PBS solution with the amplitude of 10 mV over a frequency range of 10–100 kHz using a Gamry potentiostat (PA, USA). Additionally, the ability of the developed PPy–Ag nanocomposite surface to kill the bacterial population attaching to the fabric after 24, 48, and 72 hours of exposure was visualized using a LIVE/DEAD BacLight kit (Thermo Fisher, Waltham, MA). Briefly, the exposed surface was stained with 200 μL of SYTO 8 and propidium iodide stock solution and incubated for 15 mins under a dark environment. Next, the fabric was mounted onto a glass slide with a cover slip and the fluorescent dyes were imaged using a Nikon Ti2 Eclipse microscope (NY, USA) using immersion oil. Additionally, a kill time assay was performed on the fabric surface by exposing 8-log CFU mL^−1^ of *E. coli* to the fabric surface and analysing the bacterial populations over different time points (2, 4, 6 and 24 h). The attached bacterial populations from the fabric surface were revived at each time points and plated in a TSB agar plate and the colonies were counted after overnight incubation.

### 2.7 Skin impedance measurements

To validate the stability of the PPy–Ag nanocomposite coating as a functional conductive antibiofouling dry electrode, bioimpedance measurements were performed on a skin surface. This analysis involved comparing the skin impedance measured using the developed nanocomposite-coated fabric against commercially available liquid gel-based (Ag/AgCl) electrodes. Impedance spectra were collected at a voltage of 1 V, over a frequency range from 1 Hz to 100 kHz. A pair of electrodes was placed on the skin of a healthy volunteer's arm, positioned 10 cm apart using Velcro straps. Impedance measurements were conducted over a period of 10 days, with each measurement performed in triplicate to obtain an average for each electrode.

### 2.8 Wearable measurement of ECG

The performance of the PPy–Ag nanocomposite coating as a dry electrode on a wearable garment for physiological monitoring was evaluated by monitoring ECG signals. The performance of the dry electrode was assessed by measuring ECG signals without the use of conductive gel or skin preparation and comparing them to conventional liquid gel-based Ag/AgCl electrodes. For this test the PPy–Ag nanocomposite coating on the CC was attached to a commercial compressive shirt using a Velcro attachment method. Two of these electrodes were positioned on either side of the chest, while one electrode was placed near the abdominal region, with wires connected through snap buttons. The measurements were performed using a custom made, compact and portable ECG measurement system consisting of a fully integrated single-lead ECG front-end module, an Arduino MKR 1010 WiFi microcontroller, and a 5 V Li-ion battery for power supply. The AD8232 integrated signal condition block was used as the single-lead ECG front-end module, which consists of an ultralow power analog-to-digital converter (ADC) to acquire the low-power biopotential signals. A two-pole, high gain single-stage high pass filter was integrated into the system to eliminate motion artifacts and the electrode half-cell potential. The cut-off frequency of the filter was programmed by Arduino microcontroller to remove ambient external noise. The single-lead ECG front-end module was connected to the 3.5 mm TRS audio plug cord with the cables attached to the fabric electrode by snap buttons to measure the ECG signal from the human body. The single-lead ECG front-end module was interfaced with the analog input pin of the Arduino to acquire the analog ECG signals. The signal can either be transmitted *via* WiFi or a designated cloud-based spreadsheet. The wireless remote monitoring portable ECG module was fully assembled and packaged using a dust and waterproof IP65 enclosure to form a (20 cm × 10 cm × 7 cm). ECG measurements were conducted on a healthy volunteer wearing the compressive shirt integrated with the dry electrodes at room temperature, while they were in a resting state.

### 2.9 Ethical compliance

Informed consent was obtained from all human participants involved in this study, in accordance with the principles outlined in the Declaration of Helsinki.

## 3. Results

### 3.1 Deposition of conductive Ppy–Ag nanocomposites

**Fig. 2:**
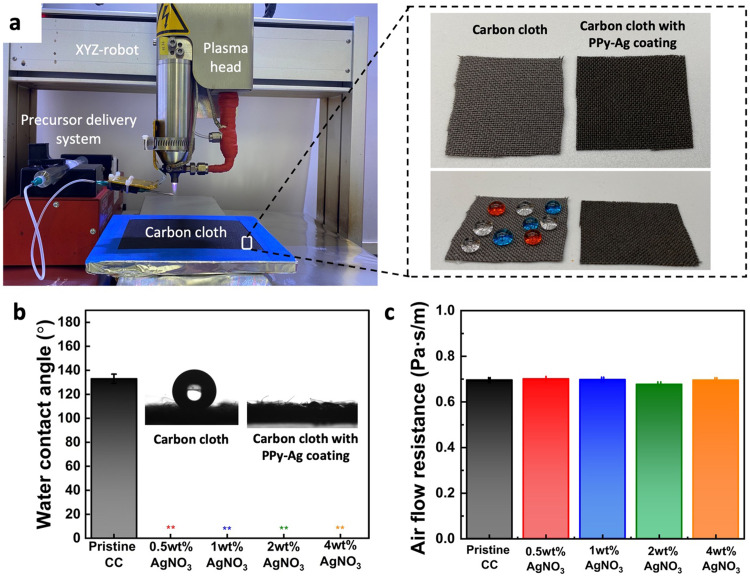
Impact of PPy–Ag nanocomposite coating on surface wettability and breathability of coated fabric. (a) Deposition setup comprising a plasma head, an XYZ-robotic system, and the precursor delivery system; images of carbon cloth before and after PPy–Ag nanocomposite coating, (b) water contact angle measurement, and (c) air flow resistance of carbon cloth (CC) before and after coating with PPy–Ag nanocomposite using varying silver nitrate (AgNO_3_) content in the precursor mixture.

The capability of CAP assisted deposition and surface modifications of functional coatings on the fabric surface was experimented using a pristine CC as the base substrate. The overall CAP deposition system with the robotic stage and the plasma glow torch with the precursor delivery system is shown in Fig. 2a. Complete coverage of a large surface area of the CC was achieved by the controlled movement of the plasma head, following a programmed toolpath trajectory across the entire fabric surface. As depicted in Fig. 2a, a noticeable change in physical appearance was observed between the pristine fabric and PPy–Ag nanocomposite-coated surface, with the modified surface exhibiting a darker coloration.

Surface wettability of the pristine CC and the PPy–Ag nanocomposite-coated surface was tested by placing color dyed water droplets on the fabric surfaces. Since carbon fibers in the pristine CC is naturally hydrophobic due to their inherent low surface energy, the dyed water droplets stayed on the fabric surface. On the other hand, PPy–Ag nanocomposite-coated surface showed a clear change in wettability as it spread quickly into the porous matrix of the fabric. This wettability characteristics was quantified by evaluating the contact angle of the water droplet on the surface. The pristine CC samples observed a contact angle of 133.0° ± 3.86 elucidating its hydrophobic nature. On the other hand, all the PPy–Ag nanocomposite coated surfaces exhibited a super-hydrophilic nature, irrespective of the AgNO_3_ precursor concentration, Fig. 2b. The super hydrophilic characteristics of PPy–Ag nanocomposite-coated surfaces can be elucidated through the theory of roughness and wettability proposed by Wenzel. According to this theory, the addition of surface roughness enhances wettability, influenced by the surface chemistry. The CAP process induces the formation of functional hydroxyl compounds in the deposited film, inherently imparting hydrophilic characteristics. However, the deposition onto the hierarchical micro and nano-textured fibrous structure of the CC further amplifies the surface's hydrophilicity leading to a super hydrophilic characteristic.

An essential consideration in the surface modification process was to preserve the inherent flexibility and breathability of the fabric for user comfort. As such the breathability characteristics of the CC fabric post PPy–Ag surface modification were assessed within a pressure range of 1–3 L min^−1^. The analysis revealed no noticeable change in air flow resistance through the fabric before and after PPy–Ag nanocomposite coating, with less than 1% variation observed (Fig. 2c). These results suggest that the PPy–Ag nanocomposite coated samples with different Ag loading concentrations did not alter the natural breathability characteristics of the fibrous CC surface.

### 3.2 Structural characterization

**Fig. 3:**
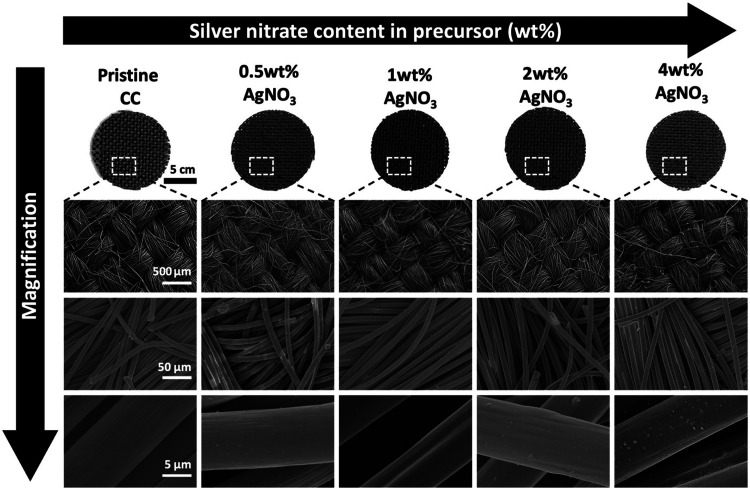
Surface morphological analysis of carbon cloth (CC) surface before and after coating with PPy–Ag nanocomposite using varying silver nitrate (AgNO_3_) content in the precursor mixture.

Surface morphological characteristics of the pristine CC and PPy–Ag nanocomposite coated fabric with different AgNO_3_ filling concentrations were analyzed using SEM analysis as shown in Fig. 3. From the micrographs, it was observed that the PPy–Ag composites had the ability to conformally deposit onto the carbon fibers without compromising the inherent gaps between the warp and welts in the fabric surface. The coated surface observed a clear smooth surface void of any agglomeration and non-uniform coating across the fabric surface. Furthermore, higher magnification of PPy–Ag deposited fabric was obtained to evaluate the change in the fiber diameter post PPy–Ag nanocomposite coating with different processing conditions. The pristine CC fibers had a smooth surface with an average diameter of 8.85 ± 1.34 μm. On the other hand, all PPy–Ag nanocomposite coated surfaces observed a small change in the average fiber diameter of 9.725 ± 2.32 μm. These results suggest that CAP-assisted deposition develops a uniform coating on individual fibers on the fabric, which is typically challenging to achieve in conventional additive deposition processes such as inkjet or screen-printing methods.^33^

### 3.3 Chemical characterization

**Fig. 4:**
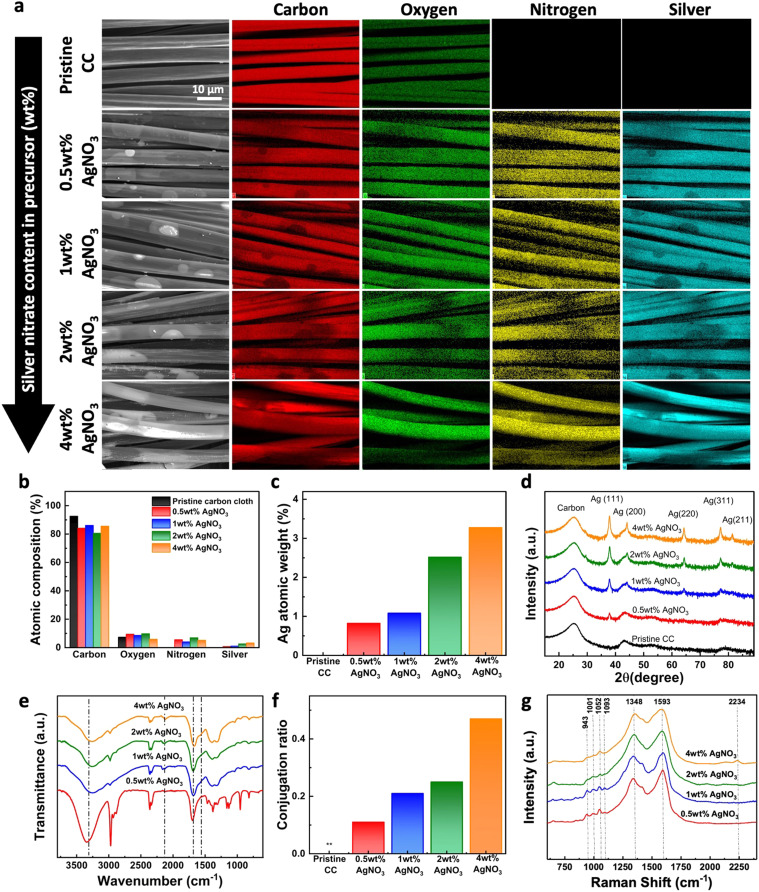
Surface chemical analysis of carbon cloth (CC) before and after coating with PPy–Ag using varying silver nitrate (AgNO_3_) content in the precursor mixture. (a) Elemental mapping and (b) composition ratio of carbon, oxygen, nitrogen, and silver on different surfaces, (c) atomic weight percentage of silver on different surfaces, (d) XRD pattern, (e) FTIR-based functional group analysis, (f) conjugation length ratio between peaks at 1660 and 1570 cm^−1^, and (g) Raman spectra for vibrational group analysis of PPy–Ag coatings.

The homogeneous distribution of the PPy–Ag nanocomposite coating on the modified fabric was assessed by performing elemental mapping on the surface for carbon, oxygen, nitrogen, and silver atoms across the deposited surfaces, as shown in Fig. 4a. As expected, pristine CC surface observed the presence of carbon and oxygen content with no trace of nitrogen and silver presence. After the PPy–Ag nanocomposite coating, all fabric surfaces, regardless of the processing conditions, exhibited a highly uniform and homogeneous distribution of nitrogen and silver atoms. Further, the % atomic compositional ratios of each element were obtained from the EDAX data. The pristine CC primarily consisted of carbon and oxygen, accounting for 92.6% and 7.4% atomic percent, respectively as shown in Fig. 4b. On the other hand, a steady increase in the silver and nitrogen content was observed upon PPy–Ag nanocomposite coating with varying AgNO_3_ precursor concentration. The high content of carbon and oxygen across all samples can be attributed to the presence of the pristine CC background material. Furthermore, the overall silver content exhibited a steady increase from 0.8% to 3.3% with an increase in AgNO_3_ precursor concentration from 0.5 wt% to 4 wt%, as illustrated in Fig. 4c.

Next, to identify the structure of the silver precursor on the deposited PPy–Ag nanocomposite surface, XRD analysis was performed. All Pristine CC and PPy–Ag nanocomposite coated fabric samples observed a broad peak at 2*θ* = 25.28° attributed to the carbon content from the background material as shown in Fig. 4d. With PPy–Ag nanocomposite deposition, a distinct change in the overall spectra was observed. New peaks located at 38°, 44.2°, 64.34°, 77.30°, and 81.44° correspond to the (111), (200), (220), (311), and (222) diffraction planes of the face-centered cubic structure of Ag.^26^ Particularly, the peak positioned in 2*θ* = 38° observed an increasing trend of the diffraction plane with increasing AgNO_3_ concentration in the precursor mixture. The observed XRD peaks were in good agreement with the JCPDS file number 04-0783. The Intensity associated with elemental AgNPs were more prominent in PPy–Ag nanocomposites coatings prepared with higher amounts of AgNO_3_ concentration in the precursor mixture. Additionally, the average crystallite size of the AgNPs concentration was observed to be in the range of 10.8–16.77 nm across all PPy–Ag nanocomposites prepared with varying AgNO_3_ precursor concentrations. Thus, XRD results indicate that the content of AgNPs in the PPy–Ag polymer matrix can be customized while maintaining consistent nanoparticle size by simply adjusting the precursor concentration in the plasma deposition process.

Next, to evaluate the formation of PPy polymeric network and level of crosslinking of PPy–Ag nanocomposite coatings with different plasma processing conditions, FTIR analysis was performed. As shown in Fig. 4e, all coated samples observed a broad shoulder peak found between 3500 to 2950 cm^−1^, which is attributed to the N–H stretching vibration band of the pyrrole ring.^34,35^ Similarly, the two peaks located at 1660 and 1570 cm^−1^ is attributed to the in-plane vibration and breathing of the pyrrole rings, indicating the retention of the PPy chemical structure.^36^ Additionally, a vibration band appearing at 2200 cm^−1^ suggests the presence of C≡N groups created from monomer fragmentation and subsequent chemical crosslinking, which was more prominent in samples containing >1 wt% AgNO_3_ in the precursor. The amplitude of the vibration band located at 1570 cm^−1^ is more prominent in PPy–Ag nanocomposites prepared with 2 wt% and 4 wt% AgNO_3_ precursor concentration implying a better polymerization and conjugation length development. The level of PPy polymerization on the CC surface was further quantified by calculating the conjugation length (ratio between two peaks located at 1660 and 1570 cm^−1^) of the deposited nanocomposite surfaces as shown in Fig. 4f.^37^ A steady increase in the conjugation ratio was observed with increasing AgNO_3_ precursor concentrations in the PPy–Ag nanocomposite coatings.

Further the vibrational modes of the deposited PPy–Ag nanocomposite coatings were analyzed to compliment the FTIR observations using Raman analysis. Fig. 4g shows the Raman spectra of the deposited PPy–Ag nanocomposite coatings with varying AgNO_3_ precursor concentrations. All PPy–Ag nanocomposite coatings exhibited two prominent and broad peaks at 1593 cm^−1^ and 1348 cm^−1^ corresponding to the symmetric C=C stretch and the ring breathing mode, respectively.^38,39^ It is interesting to note that as the concentration of AgNO_3_ increases from 0.5 wt% to 4 wt% in the precursor composition, particularly the peak at 1593 cm^−1^ tends to broaden due to the emergence of a new broad peak within the region between 1440 cm^−1^ and 1530 cm^−1^. The bands in this region have been typically assigned to the symmetric C–N stretch, antisymmetric C–N stretch, and the skeletal band of PPy. The increase in the intensity of these bands relative to the symmetric C=C stretch at 1593 cm^−1^ and the broadening of these peaks could be attributed to highly crosslinked network of PPy with inter-linkages between polymer chains. The formation of highly crosslinked polymer network can be explained by the combination of increased fragmentation, oxidation, and polymerization of pyrrole molecules and oligomers in the presence of higher concentration of AgNO_3_ during the plasma polymerization process. On the other hand, the other prominent peak located at 1348 cm^−1^ corresponding to the ring stretching mode showed considerably minimal changes with the increase in the concentration of AgNO_3_. Furthermore, four weaker peaks appearing as two doublets within the region between 920 cm^−1^ to 1100 cm^−1^ can be observed in the spectra of all four samples.^40^ While doublet at 1052 cm^−1^ and 1093 cm^−1^ represents the C–H in plane bending vibrations with neutral and doped species, the doublet bands at 943 cm^−1^ and 1001 cm^−1^ are assigned to the ring in-plane deformation modes associated with bipolaron (di-cation) or polaron (radical cation). These four bands were sharp and well-defined in the spectra of the samples prepared with 0.5 wt% and 1 wt% AgNO_3_. However, as the concentration of AgNO_3_ increased from 1 wt% to 2 wt%, these peaks begin to broaden and merge, indicating the formation of a highly crosslinked PPy network with interlinked polymer chains. These results clearly show that increasing the concentration of AgNO_3_ in the precursor mixture leads to the formation of a highly crosslinked PPy–Ag nanocomposite network on the fabric surface while retaining the chemical structure of the PPy polymer.

### 3.5 Antibacterial and biocompatibility characterization

**Fig. 5:**
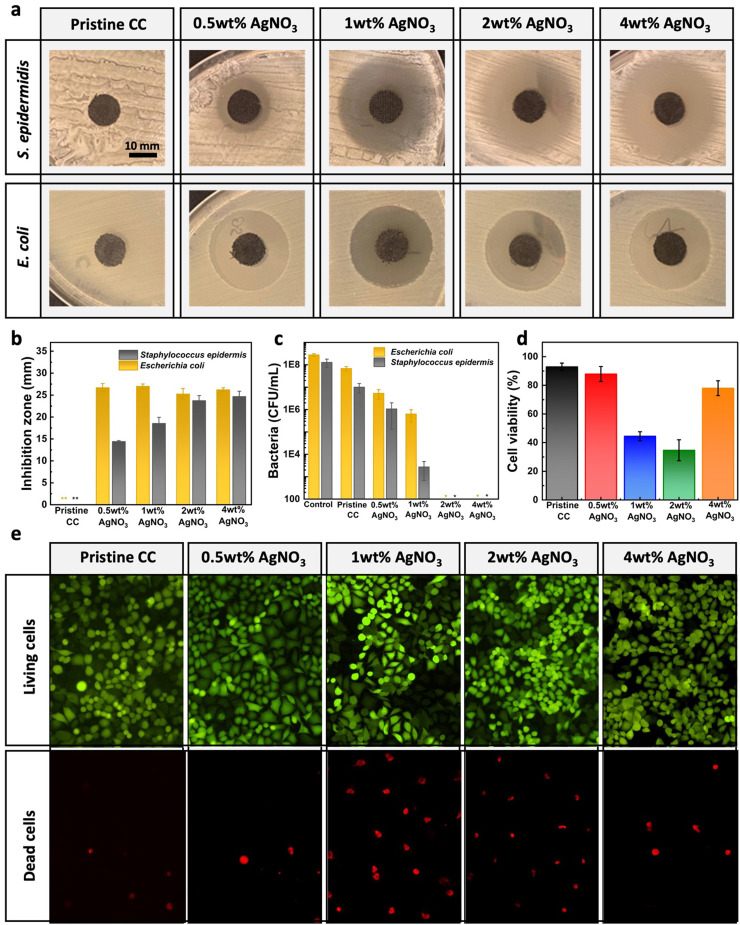
Antibacterial and biocompatibility characteristics of the PPy–Ag nanocomposite coatings. Inhibition zone based antibacterial effect with (a) plate images, (b) quantified area of inhibition, (c) antibacterial effect analyzed by contact killing analysis, (d) MTT based cell viability and (e) live/dead staining analysis of pristine carbon cloth and PPy–Ag nanocomposite coatings with varying AgNO_3_ precursor concentration.

To assess the antimicrobial activity of the deposited PPy–Ag nanocomposite coatings, two different tests were performed. First, the antimicrobial effectiveness of the pristine CC and the PPy–Ag nanocomposite-coated fabrics was evaluated by observing the zone of inhibition around the samples. As shown in Fig. 5a, no inhibition zone was observed around the pristine CC samples, indicating the absence of antibacterial properties. In contrast, all PPy–Ag nanocomposite coatings with different AgNO_3_ precursor concentrations exhibited zones of inhibition around the bacterial colonies. Both bacterial strains, *E. coli* and *S. epidermidis*, were susceptible to the developed surfaces. Notably, a higher inhibition zone was observed for *E. coli* at lower AgNO_3_ precursor concentrations compared to *S. epidermidis*, as quantitatively measured and shown in Fig. 5b. All PPy–Ag nanocomposite coatings exhibited a relatively consistent inhibition zone diameter of ∼26.6 mm against *E. coli*. In contrast, the antimicrobial activity against *S. epidermidis* notably increased with higher AgNO_3_ precursor concentrations. An increasing trend in the diameter of inhibition zones, ranging from 14 mm to ∼24.66 mm, was observed with AgNO_3_ precursor concentrations varying from 0.5 wt% to 2 wt%. The variations in antimicrobial efficacy across different bacterial strains can be attributed to the structural differences between Gram-positive and Gram-negative bacteria. Specifically, the thick peptidoglycan layer in Gram-positive bacteria imparts greater resistance to the antibacterial properties of both AgNPs and PPy.^41^

Next, to characterize the ability of the developed PPy–Ag surface to prevent bacterial attachment and subsequent biofilm formation, a contact killing test was performed. As observed in Fig. 5c, pristine CC showed no observable antibacterial effect, with bacterial concentrations revived from the surface similar to those of the stock solutions. However, with the PPy–Ag nanocomposite coating, all samples exhibited a clear reduction in bacterial attachment on the surface. In general, a noticeable antibacterial effect was observed on the fabric surface with an increase in AgNO_3_ precursor concentrations, showing complete eradication at 2 wt% and higher. These results collectively suggest that a minimum of 2 wt% AgNO_3_ is required to inhibit bacterial attachment onto the fabric surface and prevent further biofilm formation.

It was imperative to assess the biocompatibility of the PPy–Ag nanocomposite surfaces upon skin contact and determine any potential disruption of the metabolic activity of the cells in the presence of the coated fabrics. In this regard, pristine CC and PPy–Ag nanocomposite coated surface with 0.5 wt% AgNO_3_ precursor concentration showed 92.8% and 81.9% cell viability respectively, indicating excellent biocompatibility as shown in Fig. 5d. On the other hand, PPy–Ag nanocomposites prepared with 1 wt% and 2 wt% AgNO_3_ precursor concentration showed a noticeable decrease in cell viability. Progressively, further increase in the AgNO_3_ precursor concentration to 4 wt% observed increased biocompatibility of 79% and were comparable to that of control samples. The slight increase in toxicity at 1 wt% and 2 wt% AgNO_3_ precursor concentration is mainly attributed to a fast release of high amounts of loosely bound AgNPs from the PPy matrix. On the other hand, the increase in cell viability at high silver concentration is explained by the higher crosslinking degree thereby stronger entrapment AgNPs entrapment into the PPy matrix. As a result of the AgNPs entrapment, the diffusion rate of AgNPs slows down significantly reducing its cytotoxic effects. Further, live–dead staining was employed to assess the toxicity upon cell adhesion and visualize cell viability in the presence of the coated fabrics using fluorescent microscope (Fig. 5e). These results further confirmed a lower live-to-dead ratio with PPy–Ag nanocomposite coated surface with 1 and 2 wt% of AgNO_3_ in precursor concentration compared to that of other groups. Noticeably, PPy–Ag nanocomposite coating with 4 wt% of AgNO_3_ in precursor concentration showed lower dead cells similar to that of the control samples. With these results it could be deduced that the PPy–Ag coatings deposited with 4 wt% of AgNO_3_ precursor concentration showed enhanced antibacterial efficacy while simultaneously maintaining its biocompatibility characteristics. Therefore, the PPy–Ag nanocomposite coating on pristine CC deposited with 4 wt% of AgNO_3_ precursor concentration was determined to be an effective surface for further wearable electronic applications.

**Fig. 6:**
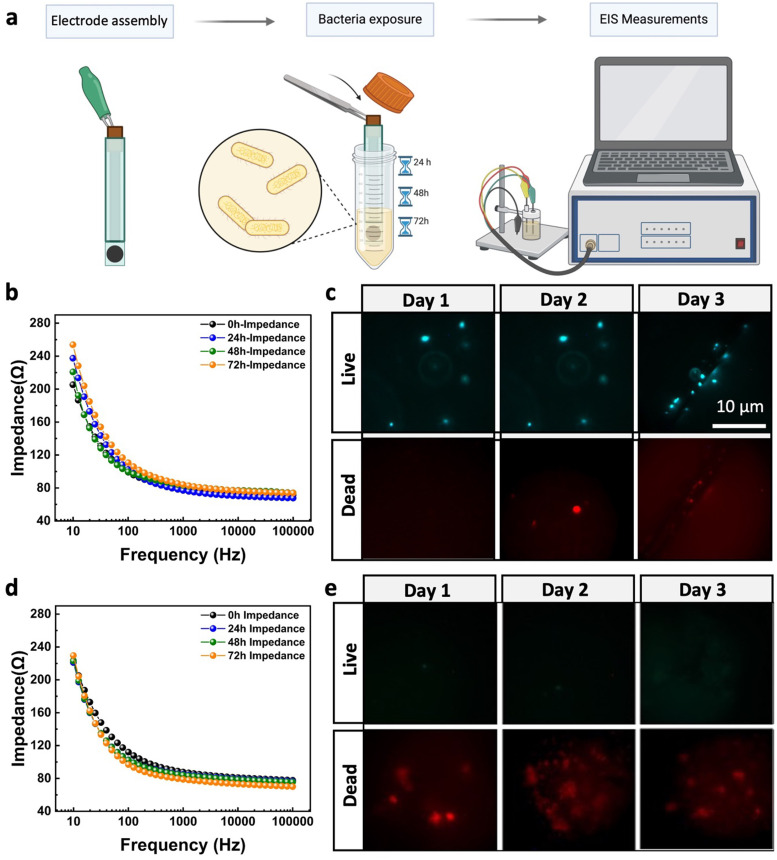
Antibiofouling analysis. (a) Illustration of the experimental process for the biofouling characterization of electrodes, (b) recorded impedance spectra of the Ag/AgCl gel-based electrode and (c) the corresponding live/dead images of the surface over the period of 3 days for Ag/AgCl gel-based electrode and (d) impedance spectra and (e) live/dead images over three days with PPy–Ag nanocomposite coating with 4% AgNO_3_ precursor concentration.

The antifouling efficacy of the optimized PPy–Ag nanocomposite coating, containing 4 wt% of AgNO_3_ precursor concentration, was assessed by monitoring changes in the electrical characteristics of the modified CC electrodes when exposed to bacterial solutions over time, as shown in Fig. 6a. This evaluation was conducted across a frequency range commonly used in bioelectrical impedance monitoring systems and wearable electronics. The pristine CC electrodes exhibited a significant increase in impedance magnitude (∼37%) within the 1–100 Hz frequency range over time as the electrodes were immersed in the bacterial culture solution (Fig. 6b). This change in impedance can be attributed to the presence and attachment of bacteria on the electrode surface, which alters the local ionic environment at the electrode/solution interface, resulting in an overall increase in electrode impedance. These findings were further validated through live/dead staining, which visualized the presence of bacteria on the electrode surface after 24, 48, and 72 hours of exposure to the bacterial culture solution. As shown in Fig. 6c, the CC surface exhibited a significant increase in live bacterial cell attachment after 3 days, with minimal dead cells present. In contrast, electrodes coated with the PPy–Ag nanocomposite showed minimal changes in impedance values, closely aligning with the intrinsic impedance values of the electrode surface across the entire frequency spectrum over the 3-day period. The stable electrical impedance characteristics of these electrodes highlight the consistent charge injection per unit area at the electrode–electrolyte interface (Fig. 6d). These results underscore the notable non-biofouling properties of the developed PPy–Ag nanocomposite coating. The live/dead staining tests further confirmed these findings, showing high levels of dead bacteria on the surface of the coated electrodes with no noticeable live bacteria throughout the 3-day study (Fig. 6e). Moreover, the equivalent circuit model for electrodes in solution, detailed in Fig. S1 and Table S1 of the ESI† document, further highlighted the electrical interfacial stability of the CC surface modified with the PPy–Ag nanocomposite coating.

To further quantify the duration required for bacteria to be killed or deactivated upon direct contact with the surface, a kill time assay was performed. This assay involved exposing the fabric surface to 8-log CFU mL^−1^ of *E. coli* and analyzing the bacterial populations at different time points (2, 4, 6, and 24 hours). As shown in Fig. S2 (ESI†), while the CC showed no signs of bacterial reduction, the PPy–Ag coated CC demonstrated complete eradication of the bacterial population after 6 hours of direct contact. These results clearly validate the efficacy of the deposited surface in eliminating bacterial attachment and subsequent biofilm formation on the fabric surface, thereby significantly reducing the effects of biofouling.

### 3.6 Physiological sensing analysis

**Fig. 7:**
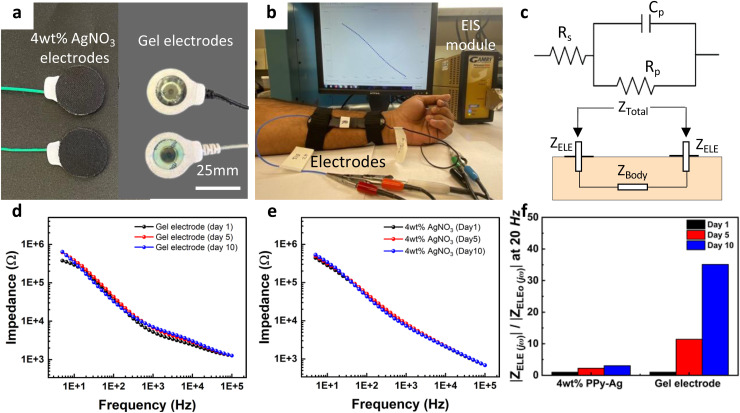
Skin impedance measurement. (a) Photograph of a pair of dry PPy–Ag coated CC electrodes and standard gel-based Ag/AgCl electrodes used for the measurements, (b) image of electrodes placed on a healthy volunteer's arm and the measurement setup, (c) equivalent electrical circuit model for the electrode–skin interface, impedance spectra of (d) gel-based electrode and (e) PPy–Ag nanocomposite coated CC electrodes, and (f) summary plot of the relative change in resistance of the *R*_ct_ element in the electrode–skin interface characteristics for each electrode.

For the implementation of the PPy–Ag nanocomposite coating as an effective surface modification to improve wearable electrode's stability and longevity characteristics, skin impedance measurements were monitored over 10 days. For this test skin bioimpedance was measured using a pair of PPy–Ag coated CC electrodes placed on the left arm of a healthy volunteer and compared against conventional liquid gel-based Ag/AgCl electrodes, as shown in Fig. 7a and b. To measure skin–electrode impedance, two electrodes were placed 5 cm apart on the inner forearm, as depicted in Fig. 7b. Elastic Velcro straps secure the electrodes firmly against the skin. A Gamry potentiostat records impedance spectra within the frequency range of 1 Hz to 100 kHz. Each measurement is taken after proper placing the electrodes on the skin to ensure electrode stability.

The mathematical representation of the skin–electrode interface adopts an equivalent RC circuit model.^42^ Distinct surface contact mechanisms in wet and dry electrodes lead to varying interfaces with the skin. The electrode–skin interfaces and corresponding equivalent circuit models (*Z*_ELE_(*jω*)) for both the wet (standard Ag/AgCl) electrode and dry electrode (PPy–Ag electrodes) are illustrated in Fig. S3 of the ESI† document. For the wet electrode, the equivalent circuit model comprises a parallel RC circuit combined with a series resistance. In this configuration, *C*_dc_ represents the double layer capacitance, *R*_es_ denotes the charge transfer resistance at the electrode/electrolyte interface (EEI), and signifies the contact resistance, which includes the resistance of the conductive gels and leads, reflecting the charge transfer at the electrolyte/skin interface (ESI). To accurately model the electrode–skin interface, the different layers of skin must be considered: the epidermis, dermis, and subcutaneous layer. The stratum corneum, due to its dielectric properties and thinness, forms a capacitive coupling between the conductive metal electrode on the skin surface and the underlying conductive tissues, represented by the capacitor *C*_s_. The resistance *R*_s_ accounts for the numerous channels, sweat glands, and hair follicles within the skin that connect these layers. The subcutaneous tissue layer, rich in blood supply, is modeled as a fixed resistance *R*_sub_ in all EIS fittings. For the dry electrode, the key structural difference is the absence of a liquid gel interface between the skin and the electrode. The galvanic connection is primarily facilitated by the natural sweat and moisture on the skin. In this scenario, the electrode behaves like a capacitor *C*_es_ at the interface, modeled as an additional RC circuit element with the resistor *R*_es_ in parallel with the capacitor *C*_es_.

Given the use of two electrodes in this skin bioimpedance measurement, the overall impedance magnitude *Z*_total_(*jω*) comprises the impedance of the first electrode *Z*_ELE_(*jω*), through the body *Z*_Body_(*jω*), and the second electrode *Z*_ELE_(*jω*), Fig. 7c. It can be expressed as:

2 |*Z*_Total_(*jω*)| = |*Z*_ELE_(*jω*) + *Z*_ELE_(*jω*) + *Z*_Body_(*jω*)|

Considering that the body impedance can be neglected in comparison to the electrode impedance, *Z*_Total_(*jω*) can be simplified to:

3 |*Z*_Total_(*jω*)| = 2 × |*Z*_ELE_(*jω*)|

In the skin impedance measurements, *Z*_Total_(*jω*) was determined using a potentiostat, and the characteristics of *Z*_ELE_(*jω*) for each electrode at every operating frequency were derived using the aforementioned equation. As depicted in Fig. 7d and e, a slight shift in impedance spectra was evident, particularly noticeable in the low-frequency region with the standard gel-based Ag/AgCl electrodes. The impedance spectra remained consistent with the PPy–Ag nanocomposite coating. The gel-based wet electrodes exhibited a significant increase in electrode–skin interface resistance, with a 40% increase in the *Z*_ELE_(*jω*)/*Z*_ELEo_(*jω*) ratio at 20 Hz, over the 10-day measurement period. In contrast, the PPy–Ag nanocomposite-coated dry electrodes demonstrated only a minimal 3% change in impedance characteristics (Fig. 7f). Additionally, EIS measurements were fitted to circuit models for both the wet (standard Ag/AgCl) and dry (PPy–Ag) electrodes. The equivalent circuit parameters for the EEI and ESI sections of the models are detailed in Fig. S3 of the ESI.† For the Ag/AgCl-based electrodes, resistance values showed a drastic change from day 0 to day 10, exceedingly over 75%, whereas the PPy–Ag dry electrodes remained stable with less than a 5% change. A similar trend was observed in capacitance values, with gel-based electrodes exhibiting a change of over 120% of the initial value after 10 days.

In the fitted models, the impedance difference primarily stemmed from the electrolyte-skin contact resistance (*R*_es_) and skin resistance (*R*_s_). It is noteworthy that while the initial specific resistance values were lower for wet electrodes, it fluctuated and increased over time. Conversely, although the dry electrodes had a higher initial resistance, it remained stable throughout the 10-day study. The instability of the gel-based electrodes for long-term measurements could be attributed to the dehydration/drying of the gel on the electrode surface and other biological factors such as biofouling. In contrast, the PPy–Ag dry electrodes ensuring a stable electrode–skin interface contact. Importantly, no signs of redness or irritation were observed after the application of the dry electrodes on the skin surface over the course of the 10-day measurements (Fig. S4 provided in the ESI†).

**Fig. 8:**
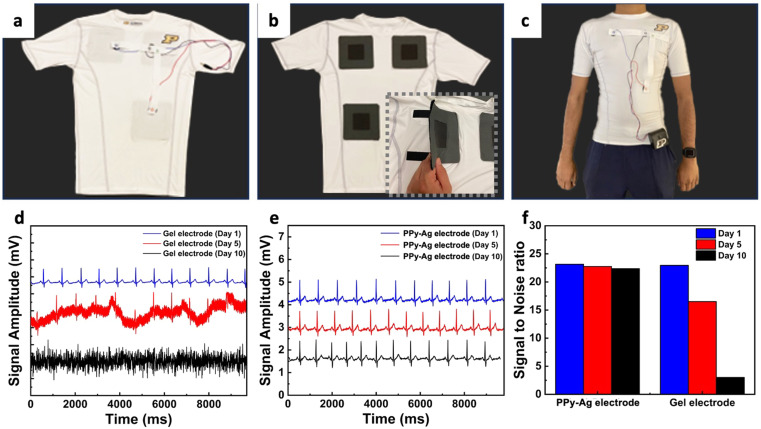
Wearable ECG monitoring system. Wearable device prototype showing the (a) outside side view, (b) inside view of the shirt with electrode interface, (c) photo of test subject wearing the integrated wearable electrode, (d) ECG measurements over time using gel-based electrode, (e) PPy–Ag nanocomposite coated electrodes and (f) SNR ratio of measured physiological signals over a 10-day period.

Finally, as a proof of concept to evaluate the utility of the PPy–Ag coating for practical use in wearable electronics, the PPy–Ag coating was applied onto flexible textile base electrodes for continuous monitoring of ECG health parameters (Fig. 8a and b). ECG measurements were collected from a shirt worn by a healthy volunteer using PPy–Ag nanocomposite surface-modified CC dry electrodes, and their performance was compared against standard gel-based Ag/AgCl electrodes (Fig. 8c). The ECG signals obtained from the gel-based electrodes and the dry electrodes are shown in Fig. 8d and 8e, respectively, and signal-to-noise ratio (SNR) performance and stability over 10 days are presented in Fig. 8f.

The gel-based electrodes exhibited significant changes in the ECG signals, with considerable background noise on day 5 and complete signal loss after 10 days of measurement, resulting in a drastic drop in SNR from 22.95 to 3.5 (84.74% change). In contrast, ECG measurements conducted with PPy–Ag dry electrodes demonstrated stable, classical ECG measurements over 10 days with a minimal SNR decrease of 3.46% over the study period. These results highlight the significant advantages of utilizing PPy–Ag nanocomposite coatings as effective antifouling agents for improved longevity in wearable electronic applications.

A summary of various PPy-based electrodes developed using different deposition strategies for diverse applications is compared against the current technology in Table S3 (ESI†). This is the first report on the development of a PPy–Ag nanocomposite surface exhibiting effective bactericidal and antifouling properties while retaining biocompatibility, used for long-term physiological monitoring applications. These results clearly demonstrate the benefits of CAP-assisted development for creating conductive polymeric surfaces, applicable to a range of uses from antibacterial coatings to electrodes. Thus, we envision that this CAP-based deposition technology could revolutionize the modification of everyday garments for the development of wearable electronic devices.

## 4. Conclusion

This study demonstrates the use of CAP processes as a scalable additive method for depositing conductive PPy–Ag nanocomposite coatings onto textile electrodes, significantly enhancing their long-term antibiofouling performance. A systematic investigation was conducted to understand the impact of precursor composition in the CAP deposition process on the surface morphology, chemical properties, electrical characteristics, structural integrity, and biofouling resistance of PPy–Ag nanocomposite coatings. The study shows that under our processing conditions, the PPy–Ag nanocomposite coatings were conformally and uniformly deposited onto individual fibers within the coated fabric. The AgNO_3_ percentage in the precursor directly influenced the amount of AgNP formed within the coating and the cross-linking degree of the PPy network. Among the various compositions, the PPy–Ag nanocomposites prepared with 4 wt% of AgNO_3_ exhibited effective bactericidal and antibiofouling properties while retaining biocompatibility, making this composition an ideal surface modification method for wearable electrodes. As a proof-of-concept, a wearable dry electrode system for continuous monitoring of ECG health parameters was developed. The results showed superior performance compared to traditional gel-based Ag/AgCl electrodes, with prolonged and stable performance over 10 days. This technology holds promise for revolutionizing the modification of everyday garments for the development of advanced wearable electronic devices.

## Data availability

The data that support the findings of this study are available from the corresponding author upon reasonable request

## Conflicts of interest

The authors declare that they have no known competing financial interests or personal relationships that could have appeared to influence the work reported in this paper.

## Supplementary Material

TC-012-D4TC00844H-s001
