# Supplementary material for: Cold atmospheric plasma deposition of antibacterial polypyrrole–silver nanocomposites on wearable electronics for prolonged performance
Source: J Mater Chem C Mater. 2024 Jul 5;12(31):11861–76. doi: 10.1039/d4tc00844h (PMC11308805; doi:10.1039/d4tc00844h)
Supplement: TC-012-D4TC00844H-s001 [file TC-012-D4TC00844H-s001.pdf]

Electronic Supplementary Material (ESI) for Journal of Materials Chemistry C.
This journal is © The Royal Society of Chemistry 2024

Supporting Information

Cold Atmospheric Plasma Deposition of Antibacterial Polypyrrole-Silver Nanocomposites on Wearable Electronics for Prolonged Performance

*Ulisses Heredia-Rivera [a,b,#], Akshay Krishnakumar [b,c,#], Venkat Kasi [a,b], Muhammad Masud Rana [b,c], Sarath Gopalakrishnan [b,c], Sina Nejati [a,b], Gagan Gundala [b,c], James Barnard [a,b], Haiyan Wang [a,b], and Rahim Rahimi [a,b,c]**

[a]School of Materials Engineering, Purdue University, West Lafayette, IN, 47907, USA

[b]Birck Nanotechnology Centre, Purdue University, West Lafayette, IN, 47907, USA

[c]School of Electrical and Computer Engineering, Purdue University, West Lafayette, IN, 47907, USA

As shown in **Figure S1**, the simplified Randle's cell is a common equivalent circuit model for electrodes in solution. In this equivalent circuit, resistors represent conductive pathways for ion and electron transfer, while capacitors and inductors represent space charge polarization regions, such as the electrochemical double layer and adsorption/desorption processes at an electrode. The simplified Randle's cell includes a solution resistance (R_s), a double-layer capacitor (C_p), and a charge transfer resistance (R_p), also known as polarization resistance. Further, the computed parameters obtained after fitting the equivalent circuit model provided in Table S1 in supporting information indicate that during the biofouling study comparing the CC electrodes with and without the antibacterial PPy-Ag coating, R_s remained stable throughout the study. This stability can be attributed to the high electrical conductivity of the test bacteria culture solution. In contrast, C_p and R_p for the uncoated CC electrodes showed a gradual increase, reaching over 38 kΩ and 3 μF change over the three-day study with continuous exposure to the bacteria culture solution. However, the PPy-Ag coated CC electrodes demonstrated relatively stable C_p and R_p values, with less than 1.5 μF and 3 kΩ of change in impedance, respectively, after three days of continuous exposure in the bacteria culture solution. This highlights the electrical stability at the electrode interface after surface functionalization, effectively reducing biofilm formation and altering ionic interactions with the electrode surface.

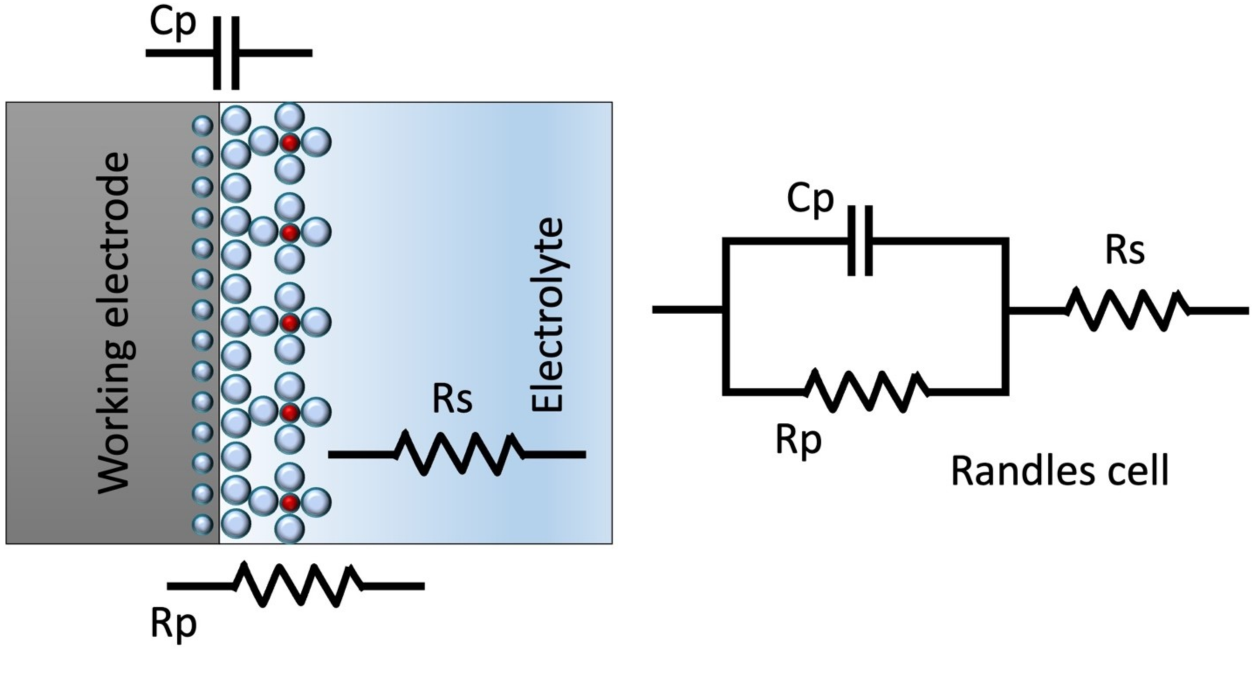

Figure S1. Simplified equivalent Randle's circuit model for electrodes in solution.

Table S1. Parameters for equivalent Randle's circuit model of electrode-solution interface for carbon cloth with and without PPy-Ag coating in bacteria culture solution over the course of 3 days.

Days	Model parameters for CC electrode			Model parameters for CC with PPy-Ag coating electrode		
	R_s (Ω)	R_p(kΩ)	C_p (µf)	R_s (Ω)	R_p(kΩ)	C_p (µf)
0	73.23	146.25	35.49	76.43	156.85	38.96
1	77.06	152.34	38.41	77.76	157.67	39.26
2	77.29	176.24	39.19	79.83	158.97	40.12
3	79.02	186.54	40.19	80.02	159.25	40.24

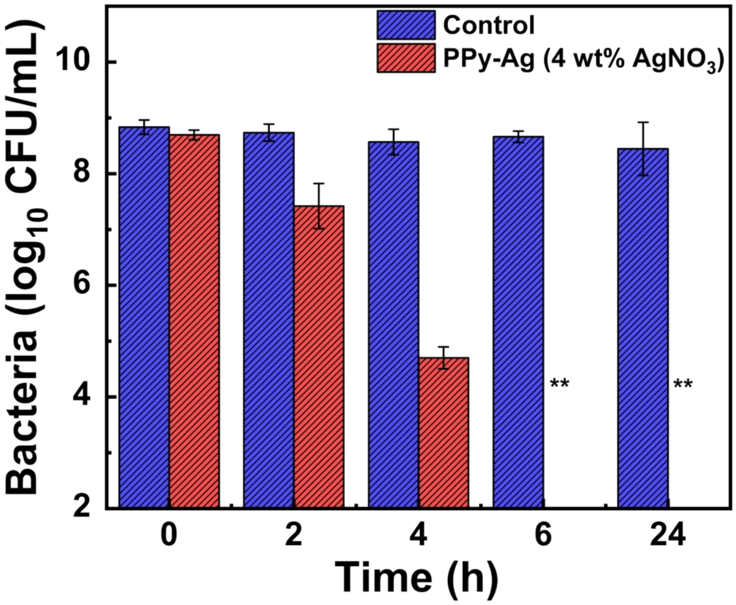

Figure S2. Kill time analysis of CC with and without PPy-Ag coating against 8-log CFU/mL of E. coli at different time points.

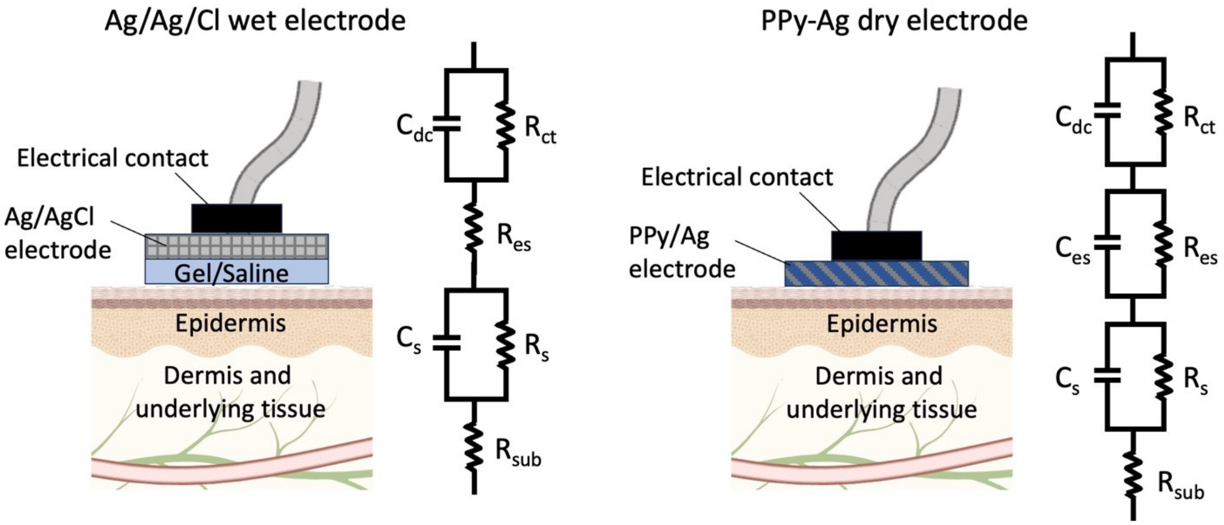

Figure S3. Schematics circuit model of electrode-skin interfaces for Ag/AgCl wet electrode and PPy-Ag dry electrodes.

Table S2. The fitting parameters for the EIS measurements across electrode-skin interfaced paired Ag/AgCl wet electrodes and PPy-Ag dry electrodes over 10 days.

**Days**	**Model parameters for Ag/AgCl wet electrode**				**Model parameters for PPy-Ag dry electrode**			
	**R_{ct} (kΩ)**	**C_{ct} (nf)**	**R_{es} (kΩ)**	**C_{es} (nf)**	**R_{ct} (kΩ)**	**C_{ct} (nf)**	**R_{es} (kΩ)**	**C_{es} (nf)**
0	2.56	6.21	39.42	-	6.08	9.38	64.36	39.18
5	4.07	9.47	62.52	-	6.14	9.54	65.10	39.70
10	6.79	14.53	69.43	-	6.34	10.10	67.85	41.00

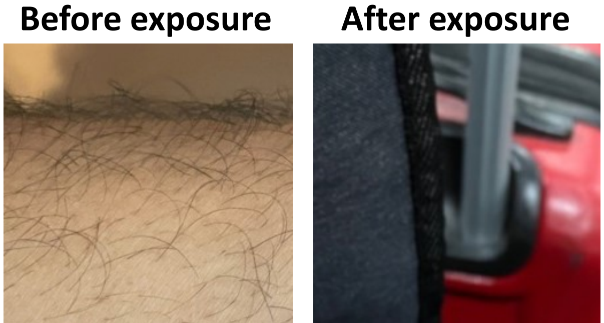

Figure S4. Image of skin surface before and after application of the PPy-Ag dry electrodes for 10 days.

Table S3. Outlook on various surfaces developed using Polypyrrole (PPy) based electrodes for various applications.

Base material	Active component	Deposition technique	Antibacterial properties	Biocompatibility	Biofouling	Application	Ref.
PPy	Ag	Chemical oxidative polymerization	*N/A*	*N/A*	*N/A*	Bioimpedance measurement	1
PPy	Ag	Chemical oxidative polymerization	*N/A*	*N/A*	*N/A*	Bioimpedance measurement	2
PDMS film	PPy-Ag	*in-situ* immersion polymerization	*N/A*	*N/A*	*N/A*	Physiological monitoring	3
PDMS film	PPy-Ag	*in-situ* polymerization	*N/A*	*N/A*	*N/A*	Body motion monitoring	4
Leather	PPy	*in-situ* immersion polymerization	Antibacterial effect against *E.coli*	Basic skin irritation	*N/A*	ECG monitoring	5
Fabric cloth	PPy-Ag	Chemical oxidative polymerization	*N/A*	*N/A*	*N/A*	Skin impedance monitoring	6
Methacrylic anhydride-based hydrogel	PPy- bovine serum albumin (BSA) introduced into poly(acrylamide-co-acrylic acid) matrix	*in-situ* polymerization	*N/A*	Biocompatibility with HeLa cells	*N/A*	ECG monitoring	7
Cotton fabric	Polydopamine decorated PPy	*in-situ* dip polymerization	*N/A*	*N/A*	*N/A*	Human motion detection	8
PET with Au coating	PPy	Electrochemical polymerization	Antibacterial effect against *E.coli*	Biocompatibility MRC-3 fibroblasts	*N/A*	*N/A*	9
**Carbon fabric**	**PPy/Ag**	**Cold Atmospheric plasma-based**	**Antibacterial effect against *E.coli* and S.**	**Biocompatibility HCT-8 epithelial cells**	**3-day antifouling test against**	**ECG monitoring for 10 days**	**This work**

	deposition	*aureus*	*E.coli*

References

1 G. Telipan, L. Pislaru-Danescu, I. Ion and V. Marinescu, in *2020 International Semiconductor Conference (CAS)*, 2020, pp. 23–26.

2 L. PÎSLARU-DĂNESCU, V. STOICA and G. TELIPAN, *Electrotehnica, Electronica, Automatica*, 2021, **69**, 51–58.

3 D. Wang, X. Zhou, R. Song, C. Fang, Z. Wang, C. Wang and Y. Huang, *Chemical Engineering Journal*, 2021, **404**, 126940.

4 J. Peng, B. Wang, H. Cheng, R. Yang, Y. Yin, S. Xu and C. Wang, *Compos Sci Technol*, 2022, **227**, 109561.

5 K. Zhang, N. Kang, B. Zhang, R. Xie, J. Zhu, B. Zou, Y. Liu, Y. Chen, W. Shi, W. Zhang, W. Huang, J. Wu and F. Huo, *Adv Electron Mater*, 2020, **6**, 2000259.

6 G. Telipan, L. Pîslaru-Dănescu, E.-M. Lungulescu, I. Ion and V. Marinescu, *Applied Sciences*, , DOI:10.3390/app11094168.

7 J. Xu, H. Zhang, Z. Guo, C. Zhang, H. Tan, G. Gong, M. Yu and L. Xu, *Int J Biol Macromol*, 2023, **230**, 123195.

8 Z. Yu, Z. Zhu, Y. Wang, J. Wang, Y. Zhao, J. Zhang, Y. Qin, Q. Jiang and H. He, *Cellulose*, 2023, **30**, 5355–5371.

9 Y. Wu, D. Xiao, P. Liu, Q. Liao, Q. Ruan, C. Huang, L. Liu, D. Li, X. Zhang, W. Li, K. Tang, Z. Wu, G. Wang, H. Wang and P. K. Chu, *Research*, 2023, **6**, 0074.
